# Antibiotic resistance assessment of *Acinetobacter baumannii* isolates from Tehran hospitals due to the presence of efflux pumps encoding genes (*adeA* and *adeS* genes) by molecular method

**DOI:** 10.1186/s13104-020-05387-6

**Published:** 2020-11-19

**Authors:** Batool Basatian-Tashkan, Mohammad Niakan, Mansoor Khaledi, Hamed Afkhami, Fatemeh Sameni, Shahriar Bakhti, Reza Mirnejad

**Affiliations:** 1 Department of Microbiology, Faculty of Medicine, Shahed University, Tehran, Iran; 2 Molecular Biology Research Center, System Biology and Poisoning Institute, Baqiyatallah University of Medical Sciences, Tehran, Iran

**Keywords:** *Acinetobacter baumannii*, Efflux pump, adeA gene, adeS gene, Antibiotic resistance

## Abstract

**Objective:**

*Acinetobacter baumannii* (*A. baumannii*) has caused many problems in nosocomial infections. Efflux pumps are considered as one of the most important mechanisms of resistance in this bacterium and have the ability to excrete toxic substances such as antibiotics out of the cell.

**Results:**

In this study, 60 isolates of *A. baumannii* were collected from patients in several hospitals in Tehran, Iran. After diagnosis using standard biochemical methods, the pattern of antibiotic susceptibility was determined using the disk diffusion method according to CLSI guidelines. The adeA and adeS genes were identified by PCR method. The highest resistance to Piperacillin and the lowest resistance to Gentamicin were observed (100% compared to 48.4%). 6.6% of the isolates had only adeA gene and adeS gene was observed in 8.4% of isolates and both genes were detected in 73.4% of the samples. Despite the high resistance of t *A. baumannii* o antibiotics and due to the high frequency of genes of adeA and adeS efflux pumps in *A. baumannii* isolates, it can be concluded that these efflux pumps may play an important role in resistance of this bacterium. By determining the pattern of antibiotic the resistance before treatment, the resistance of this pathogen can be prevented in societies.

## Introduction

Nosocomial infections are known as one of the crucial problems for public health [1]. Most of these infections are caused by Gram-negative bacilli [2]. One of the most important causes of nosocomial infections is *A. baumannii* that is Gram-negative Coccobacilli and aerobic and prefer humid environments for a living [3, 4]. The most important pathogen of this genus is, *A. baumannii* which can cause a wide range of diseases and nosocomial infections such as pneumonia, septicemia, urinary tract infections, skin and wound infections, endocarditis, and meningitis [5, 6]. Infection with this bacterium is increasing in people with immunodeficiency or cancer [7]. One of the main problems caused by this bacterium is the development of high antibiotic resistance [8]. One of the mechanisms that this bacterium uses to resist various antibiotics is the use of efflux pumps. By using efflux pumps, the *A. baumannii* can direct antibiotics outwards and prevent antibiotics from affecting the bacteria [9]. Due to the high prevalence of efflux pump genes especially AdeA and AdeB that are the major discovered efflux pumps in *A. baumannii*, this may play a significant role in the antibiotic resistance of *A. baumannii* isolates. Identifying the antibiotic susceptibility pattern is essential to prevent the prevalence of antibiotic resistance. For this purpose, this study was performed by determining the antibiotic susceptibility in clinical trials of *A. baumannii* collected from several hospitals in Tehran.

## Main text

### Materials and methods

#### Patients and sampling

In this study, 60 *A. baumannii* isolates were collected from 100 different samples including chips, blood, urine, wound culture, respiratory secretions, catheters, spinal fluid, and pleurisy during the 9 months from August 2018 to May 2019 from different departments of Milad Hospital, Baqiyatallah and the Rasool-Akram hospital of Tehran was identified.

1. Isolation and identification of *A. baumannii* strains
  Prevalent biochemical tests and methods have been used to identify and confirm *A. baumannii* strains [10].
  The specimens were inoculated on blood agar (Merck) and MacConkey agar (Merck) medium and incubated for 24 h at 37 °C. Conventional biochemical methods such as oxidase, citrate, urea urease, malonate consumption, oxidation and fermentation of sugars, motility, and indole production were done to identify *A. baumannii* [11].

2. Confirmation of *Acinetobacter baumannii* by PCR
  To confirm the *Acinetobacter baumannii*, the gene *bla*_OXA-51-like_ was examined by PCR similar to the method performed by Jia et al. [12]. Because it has been made clear that, there is OXA-51–like gene in Acinetobacter baumannii isolates instinctively [13].

#### Antimicrobial susceptibility test

This test was performed using the Kirby Bauer method using 9 disks including Imipenem (10 μg), meropenem (10 μg), Gentamicin, Piperacillin, Ampicillin-Sulbactam, Ceftazidime, Amikacin, Tetracycline, and Ciprofloxacin. The 2019 CLSI was interpreted [14]. The *Acinetobacter baumanii ATCC19606* was used as quality control for this test.

#### DNA extraction

DNA extracting was performed by DNA extraction kit (Bioneer Company Korea, Cat. No. K-3032-2-) was used.

#### Identification of adeA and adeS gene via PCR

PCR was performed to screen for *adeA* and *adeS* genes. The primer sequences used are shown in Table 1. The PCR program For amplification was: an initial denaturing step, 5 min at 94 *°*C, 30 cycles of the 30 s at 94 *°*C, primer connection for *adeA* at 55.5 °C and For *adeS* at 54.5 °C for the 30 s, 90 s at 72 *°*C, and 5 min as a final extension at 72 *°*C were performed. The PCR products were analyzed through a 2% agarose gel containing Syber safe and were detected using a gel documentary device.

**Table 1:** Frequency distribution of *Acinetobacter baumanii* isolated from patients according to the type of clinical sample and hospital

Sample type	Baqiyatallah	Rasool-Akram	Milad	N (%)
Tracheal	2	1	1	4 (6.6)
Wound	6	3	4	13 (21.8)
Urine	3	2	2	7 (11.8)
Blood	6	5	5	16 (26.6)
Catheter	1	1	2	4 (6.6)
	7	3	6	16 (26.6)

The primers designed in this study are as follows: VIM-1 5′-TGGTTGTATACGTCCCGTCA TGTGTGCTGGAGCAAGTCTA-3′, IMP-15′-TAACGGGTGGGGCGTTGTTCCT CGCCCGTGCTGTCGCTATGAAA-3′ and The third primer for OXA-51 was described previously [11].

Primers for *adeA* and *adeS* genes:

*adeA* Forward 5′-TTGATCGTGCTTCTATTCCTCAAG-3′

*adeA* Reverse 5′-GGCTCGCCACTGATATTACGTT-3′ [15]

*adeS* Forward 5′- TGCCGCCAAATTCTTTATTC-3′

*adeS* Reverse 5′- TTAGTCACGGCGACCTCTCT-3′ [16].

#### Statistical analysis

Questionnaire information and the results of phenotypic and genotypic experiments were analyzed using SPSS software, version 23, by the Chai test and Fisher’s exact test.

### Results

In this study, 60 bacterial isolates were collected as *Acinetobacter baumanii* from three hospitals in Tehran. The frequency distribution of *Acinetobacter baumanii* Isolates according to the sample is shown in Table 1.

#### Antibiogram results by agar disc diffusion method

Out of 60 *A. baumannii* isolates, all of them were resistant to Piperacillin, and this rate was as follows in other antibiotics: Ceftazidime 98.4%, Amikacin 96.6%, Tetracycline 91.6%, Ampicillin-Sulbactam 65%, Meropenem 63.4%, Ciprofloxacin 61.6%, Imipenem 50%, and Gentamicin 48.4%, which had the considerable effect and Piperacillin and Ceftazidime showed the most resistance levels (Table 2).

**Table 2:** Antibiotic resistance pattern of *Acinetobacter baumanii* isolates

Antibiotic	Resistant N (%)	Intermediate N (%)	Susceptible N (%)
Piperacillin	60 (100)	0 (0)	0 (0)
Ceftazidime	59 (98.4)	1 (1.6)	0 (0)
Amikacin	58 (96.6)	0 (0)	2 (3.4)
Tetracycline	55 (91.6)	2 (3.4)	3 (5)
Ampicillin-Sulbactame	39 (65.0)	2 (3.4)	19 (31.6)
Meropenem	38 (63.4)	1 (1.6)	21 (35)
Ciprofloxacin	37 (61.6)	3 (5.0)	20 (33.4)
Imipenem	30 (50.0)	0 (0)	30 (50.0)
Gentamicin	29 (48.4)	0 (0)	31 (21.6)

#### Results of genotypic tests

The presence of the *OXA*-*51* gene in the *Acinetobacter baumannii* was investigated. All samples in the PCR method had this gene. The frequency distribution of the *adeA* and *adeS* genes in the PCR method was also examined. The frequency of adeA and adeS genes in the samples were 6.6% and 8.4%, respectively calculated by PCR method. In 44 (73.4%) of the samples, these two genes were present together and in 7 (11.6%) of the samples, none of the adeA and adeS genes were present.

#### Results of the relationship between antibiotic resistance and efflux pump genes

According to the results, it can be concluded that the expression of *adeA* and *adeS* genes are related to the Tetracycline, Ciprofloxacin, Gentamicin, and Amikacin resistance (Table 3).

**Table 3:** Antibiotic resistance in acinetobacter bacterial isolates

Antibiotic	Resistant N (%)	Resistant. *ade A* gene (%)	Resistant. *ade S* gene (%)
Amikacin	58 (96.6)	48	49
Tetracycline	55 (91.6)	36	36
Ciprofloxacin	37 (61.6)	29	29
Gentamicin	29 (48.4)	48	49

Studies show that in addition to efflux *adeABC* pumps, other efflux pumps also have a leading role to play in developing resistance to these antibiotics, and the simultaneous presence of these efflux pumps increases resistance. Although other factors contribute to this antibiotic resistance, efflux pumps play the most crucial role in creating resistance to these antibiotics [17].

### Discussion

Nosocomial infections have become one of the main problems in treatment. The most common cause of nosocomial infections is Gram-negative bacteria. *Acinetobacter baumannii* is an opportunistic hospital pathogen that causes a wide range of nosocomial infections. Due to the indiscriminate use of broad-spectrum antibiotics by people, we are witnessing high antibiotic resistance caused by this bacterium. The high antibiotic resistance of this bacterium is associated with the proliferation of multiple antibiotic resistance genes. Various studies have shown that *Acinetobacter baumannii* is resistant to most Beta-lactam antibiotics and Quinolones, and its resistance to Aminoglycosides is increasing. In this study, the most effective antibiotics used against *Acinetobacter baumannii* were Gentamicin and Imipenem, which had 48.4% and 50% resistance, respectively, while the highest resistance was to Piperacillin (100%), Ceftazidime (98.4%), and Amikacin (96.6%) and Tetracycline (91.6%).

In the present study, the resistance of *Acinetobacter baumannii* to at least one antibiotic was seen in three groups or more in all isolates. Therefore, the frequency of multi-drug resistance in *Acinetobacter baumannii* isolates was 100%, but the frequency of *Acinetobacter baumannii* with multiple resistance in other studies reported was reported to be 50 to 100% variable [12]. High MDR levels in *Acinetobacter baumannii* studied in Farsiani et al. Studies in Iran and Rynga et al. In India were reported to be 97% and 85%, respectively [18, 19], which could be due to abuse of antibiotics.

In the Noori et al. study, All isolates had 100% resistance to Ceftazidime, Ciprofloxacin, and Piperacillin, and the frequency of *the adeS* gene was 91% which in comparison to Ciprofloxacin *adeS* resistance was different [20]. According to the results of the present study, we saw a decrease in antibiotic resistance to ceftazidime and ciprofloxacin and the same resistance to piperacillin was reported.

Research by Rahbarnia et al. Found that the resistance to Ciprofloxacin was 95%, Imipenem 82%, and Gentamicin 35%. Also, the prevalence of MDR and XDR in the studied strains was 76% and 30%, respectively, which compared to the present study, higher resistance to Ciprofloxacin and Imipenem has been reported [21]. This reason for the increase in antibiotic resistance may be related to different geographical areas in Iran.

In Abdar et al. study, the resistance to Meropenem and Ceftazidime was reported to be 71% and 93%, respectively, which is very similar to the present study [22].

According to Fallah et al., The antibiotic resistance of *Acinetobacter baumanii* isolates is as follows: 95.4% to Ceftazidime, 91.7% to Meropenem, and 92.6% to Ciprofloxacin, which is reported to have a higher resistance to Meropenem and Ciprofloxacin than the present study [23]. The reason for the decrease in antibiotic resistance in recent years in Iran can be due to changes in antibiotic treatment policies and the use of antibiograms before prescribing antibiotics.

According to a report by Al-Agamy et al., 100% of *Acinetobacter baumanii* isolates were 85% resistant to Ciprofloxacin and 70% to Imipenem [24]. Compared to the present study, we saw a decrease in resistance to both antibiotics.

According to the study of Angoti et al., The resistance to Ciprofloxacin is 99%, Ceftazidime, Meropenem, and Imipenem 98%, Gentamicin 77%, Amikacin 48% which is different from the present study and also the prevalence of *adeA* gene in 61 samples 88.5% was reported [25]. Based on the results of a recent study, we have seen a decrease in antibiotic resistance in recent years in the treatment of patients.

In a study by Boral et al. [26], antibiotic resistance for Ciprofloxacin, Imipenem, Ampicillin/sulbactam, Ceftazidime, and Amikacin was observed to be 100%, 99.4%, 99.4%, 99.4%, and 91.8%, respectively. That is an increase in reported antibiotic resistance compared to the present study.

In a study by Ranjbar et al., The antibiotic resistance to Ceftazidime, Ciprofloxacin, Piperacillin, Gentamicin, Amikacin, and Ampicillin/Sulbactam was reported to be 97.5%, 96.3%, 95.1%, 92.1%, 87.2%, and 76.1%, respectively [27]. One of the reasons for the difference in antibiotic resistance compared to the recent study is the diverse geographical areas for sampling patients.

In a study by Jia et al. China, resistance to Piperacillin and Ceftazidime was reported 92.2% that was similar to this study [12].

The frequency of Tetracycline resistance in the studied isolates was 91.6%. The results of studies conducted in Iran were similar to the results of studies of Sarhaddi et al. [28] that were 96.3% and compared to the study of Nemec et al. Antibiotics were 62% higher [29].

One of the resistance mechanisms in the *Acinetobacter baumanii* is the presence of efflux pumps. These pumps cause the leakage of antibiotics and a wide range of substances out of the bacteria, creating multidrug resistance. Three systems, *AdeFGH*: *RND*, *AdeIJK*, and *AdeABC*, have been identified in the *Acinetobacter baumanii*, among which *AdeABC* is most involved in the MDR *Acinetobacter baumanii* [30]. Although high levels of resistance do not occur only as a result of multi-drug efflux pumps, the expression of their genes among isolates with high antibiotic resistance cannot be ignored. Therefore, it is necessary to identify resistance systems, including efflux pumps.

In the tracking section of efflux pumps encoding genes, 80% (48 samples) of 60 *Acinetobacter baumanii* isolates had *adeA* gene and 81.66% (49 samples) isolates had *adeS* gene, which compared to other studies conducted in Iran, Japanese race et al. [27] (*adeA* 100%), Beheshti et al. [31] (for all gene pumps *AdeABC* 100%) and Ardabili et al. [32] (*adeS* 100%) were lower.

Compared to studies conducted abroad by Jia et al. In China [12], 79.6% of the *Acinetobacter baumanii* isolates had the *adeA* gene and 80.6% had the *adeS* gene, also in the study conducted by Nemec et al. in France [29] 81.9% of the isolates had *adeA* and *adeS* genes that were similar to the results of our study but compared to the research by Kor et al. [33] that the frequency of *adeA* gene in isolates was 62.7% that was higher. The reason for the differences in different studies could be due to differences in the patterns of antibiotic use, the type of clinical sample, the number of samples studied, sampling method, environmental factors and the different geographical distribution of these genes.

## Conclusion

A significant increase in antibiotic resistance is one of the main problems in the treatment of infections caused by *Acinetobacter baumannii*. Efflux pumps play a key role in the development of multiple resistance to antimicrobial drugs. It is important to evaluate the presence of efflux pump genes in preventing the spread of antibiotic resistance and to suggest an appropriate treatment model for patients infected with this bacterium. So the high prevalence of genes encoding efflux pumps in this bacterium is one of the important factors in the spread of antibiotic resistance between isolates in different geographical regions. Of course, the role of other factors and mechanisms involved in the development of *Acinetobacter baumannii* resistance should not be ignored.

## Limitations

Our limitation in this research project was the lack of sufficient funding. If there is more budget, it can be further developed and more methods can be added.

## Data Availability

All relevant data are included in the manuscript.

## Abbreviations

PCR: Polymerase chain reaction
CLSI: Clinical and Laboratory Standards Institute
MDR: Multi drug resistance
ATCC: American type culture collection
RND: Resistance-nodulation-division
IMP: The genes imipenemase
VIM: Verona integron-encoded metallo-beta-lactamases
